# A Qualitative Exploration of the Help-Seeking Behaviors of Students Who Experience Psychological Distress Around Assessment at Medical School

**DOI:** 10.1007/s40596-017-0701-9

**Published:** 2017-03-31

**Authors:** Rachel I. Winter, Rakesh Patel, Robert I. Norman

**Affiliations:** 1 University of Leicester, Leicester, UK; 2 University of Nottingham, Nottingham, UK

**Keywords:** Medical students, Mental health, Support, Examination

## Abstract

**Objective:**

Medical students are at high risk of experiencing psychological distress at medical school and developing mental ill-health during professional practice. Despite efforts by faculty to raise awareness about this risk, many students choose to suffer in silence in the face of psychological distress. The aim of this study was to explore drivers that prompted help-seeking behavior and barriers that prevented individuals prioritizing their well-being around the time of high-stakes assessment at medical school.

**Methods:**

Semi-structured interviews were conducted with fifty-seven students who failed high-stakes assessment at two UK medical schools, exploring their experience of academic difficulty and perceptions about causes. A thematic analysis of twenty transcripts that met inclusion criteria was completed to identify key factors that influenced participants’ decisions around seeking help for their psychological distress, and in some cases, mental health problems. Twenty participants who specifically described a deterioration in their mental health around the time of assessment were included in this study.

**Results:**

Barriers to seeking help in these instances included: normalization of symptoms or situation; failure to recognize a problem existed; fear of stigmatisation; overt symptoms of mental distress; and misconceptions about the true nature of the medical school, for example beliefs about a punitive response from the school if they failed. Drivers for seeking help appropriately included: building trust with someone in order to confide in them later on, and self-awareness about the need to maintain good mental health.

**Conclusion:**

There are various drivers and barriers for students’ help seeking behaviors when experiencing psychological distress around the time of assessment, particularly self-awareness about the problem and prioritisation of well-being. Students who fail to recognize their own deteriorating mental health are at risk of academic failure and medical schools need to develop strategies to tackle this problem in order to protect these students from harm.

Medical students report high rates of depression, anxiety, and suicidal thoughts [1–4] and tend to be more vulnerable to developing mental illness compared to age-matched peers [4] or the general population [5]. Despite these apparent differences between medical students and others, there is evidence suggesting the emotional status of individuals at the start of medical school is the same or similar to that of the general population [6]. There are many proposed triggers of psychological distress among medical students to explain the emergence of any difference after starting the course, such as early exposure to death and dying [7] and greater burden of work and high-stakes assessments [8]. Although the medical school experience presents these various challenges, the majority of students complete the course without difficulty and, in some cases, with excellent results. However, the same is not true for all, and the experience may lead to significant psychological distress among particular groups of students [9, 10].

The development of psychological distress into mental illness may have serious implications for the student who experiences recurrent academic failure, and for the public who the doctor may eventually care for in practice. For the student, there is an immediate risk to personal health and well-being as well as a long-term risk of mental health problems in professional practice [2]. Possible consequences for the public include a potential threat to patient safety, albeit over the long-term, which is difficult to accurately quantify [11]. There is a responsibility on medical schools to prevent these potential and plausible outcomes in light of this evidence; therefore, the need to better understand the role and influence of stressors such as high-stakes assessment on triggering psychological distress is a priority.

Although there are a number of studies that describe various barriers to students attempting to access treatment [12–16], there remains little understanding around the help-seeking ability of individuals to actually get help when experiencing acute psychological distress or deteriorating mental health. This study explored the help-seeking behavior of medical students who experienced psychological distress or deteriorating mental health prior to failure at high-stakes assessment. The research question for this study was: “What are the drivers and barriers that influence students’ help-seeking behaviors when experiencing psychological distress or deteriorating mental health around the time of high-stakes assessment?”

## Methods

### Participants and Setting

This study is part of a larger program of research conducted across two medical schools in the UK seeking to better understand the lived experience of failure at high-stakes assessment and the implications for academic, personal, and professional support for students in remediation [17, 18]. The complexity of the lived experience for students, as well as the academic reasons for failure, emerged from a primary analysis of the data [17]; however, a number of health and personal problems were also described, prompting an in-depth secondary analysis of the original data for presentation in this paper.

The participants were students attending medical schools with two distinct undergraduate and graduate-entry courses. Both medical schools provided a 5-year program with a traditional lecture-based teaching format in the early years, and greater exposure to clinical teaching in the workplace during later years. The 4-year graduate-entry program varied between the two schools. One school offered a problem-based learning approach, whereas the other offered an accelerated lecture-based version of the undergraduate course. The final examination taken by students at both universities included a combination of two single best answer format written papers and a clinical objective structured clinical examination (OSCE) circuit. For students who failed their final high-stakes clinical examination at the end of any of the courses, including the re-sits, there was an opportunity for them to undertake a period of formal remediation and extend their time on the course. Students who were entered into remediation were emailed an invitation to participate in the study and describe their experience of studying on the course.

### Data Collection

All participants completed an in-depth semi-structured interview (available on request from the authors) intended to explore their journey through medical school with a particular focus on their experiences of failure at high-stakes assessment [17, 18]. There were no specific questions about psychological distress or mental health problems to minimize the impact of interviewer bias on the data collection process. Nevertheless, the impact of failure on well-being was explored in every case and direct questions were used to explore psychological distress or mental health problems as participants’ volunteered examples around the time of assessment. For example, when the student experience suggested the presence of “low mood” or “suicidal ideation,” direct questions probed the nature of these problems, including any impact on personal well-being; help-seeking behavior including the recognition of problem in the first place; relationships with others; decisions about study and academic outcomes. All participants were asked to consider, in hindsight, what they would have done differently, what help they might have sought, and who from, in order to get a rich description of the context in which drivers or barriers for students influenced their general help-seeking behavior at medical school. The interviews lasted up to 90 min and were digitally recorded with the permission of the student.

**Fig. 1:**
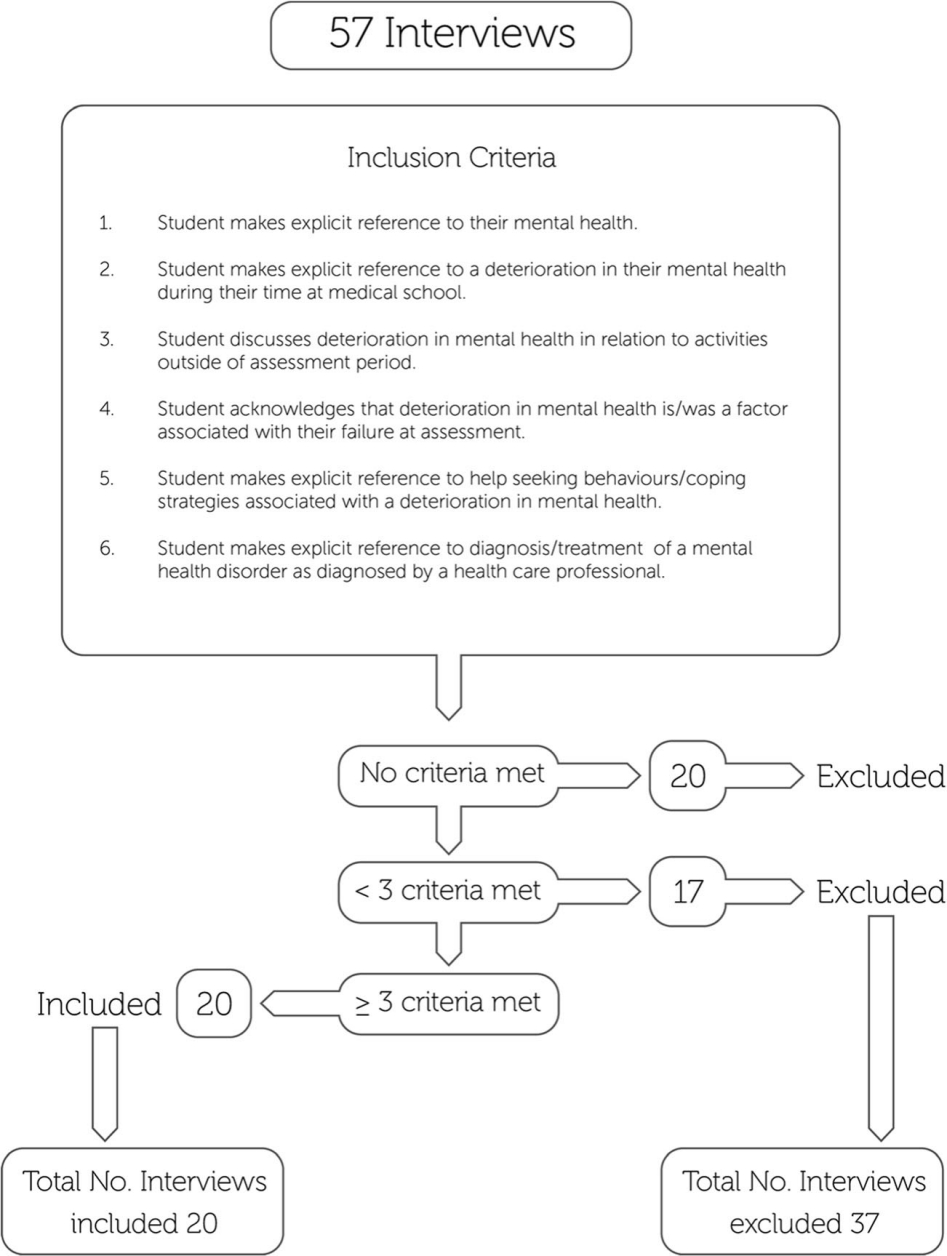
Inclusion criteria applied to transcripts

The process for identifying participants whose experiences would be suitable for inclusion in this study is described in Fig. 1. All participants described some reference to an adverse reaction following their failing experience; however, only interviews from participants whose personal stories included three specific and separate instances of psychological distress or deterioration were analyzed in greater detail for the purposes of this study. All interviews included were anonymized and transcribed verbatim.

### Data Analysis

Thematic analysis (TA) was used to explore and code the raw data from the interviews. Unlike discourse analysis or interpretive phenomenological analysis, thematic analysis is not confined to any pre-existing theoretical framework and can be used within different frameworks and in different ways [19]. TA can be explicitly informed by pre-existing theories (deductive), while allowing the generation of new theories from the data studied (inductive) [20]. Using an inductive TA approach for this study, theme development was directed by the content of the data and allowed the identification of potentially previously unreported themes.

RW and RP began the data analysis by reading all transcripts included in the study to generate initial codes within the data. RW and RP re-read the first eight transcripts to organize the initial codes into themes. Themes were compared across transcripts to generate a framework of higher-order and sub-themes. The framework was discussed and refined within the research team (RW, RP, RN). RW, RP, and RN applied the framework to code all data from the remaining transcripts and revised the framework as necessary to incorporate any appropriate new or emergent themes.

## Results

Fifty-seven interviews were conducted between 2009 and 2014 across the two medical schools, and interviews from 20 participants met the inclusion criteria for this study (Fig. 1). The demographic details of participants are presented in Table 1.

**Table 1:** Demographics of participants

Demographics	Number
Male	12
Female	8
Primary or secondary in the UK prior to entry into medical school	13
International student	7
Graduate-entry student	6

*n* = 20

**Fig. 2:**
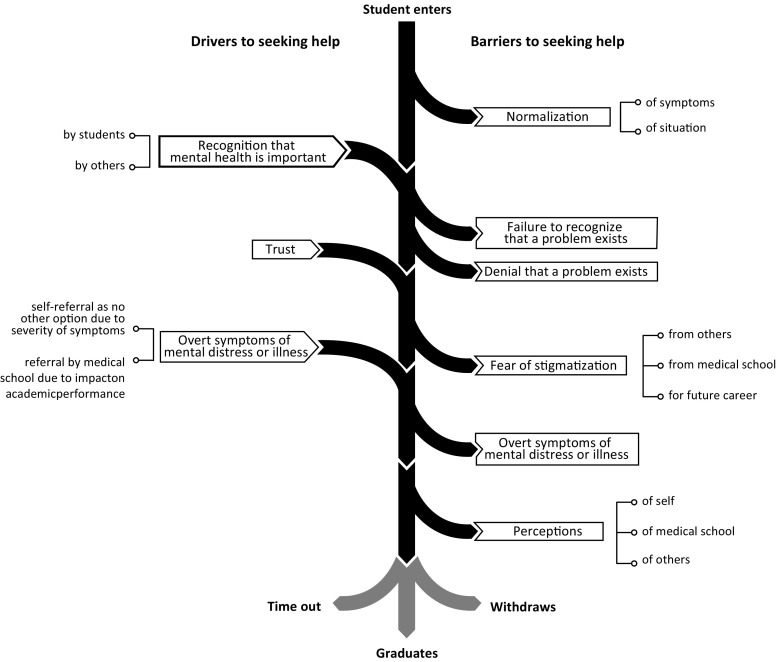
Drivers and barriers identified through thematic analysis

The emergent themes identified from this study are illustrated in Fig. 2, which presents the individual barriers that prevented deteriorating mental health from being prioritized by students alongside the drivers that prompted some students to appropriately seek help when experiencing psychological distress or deteriorating mental health. Some factors were a driver in some instances but a barrier in other cases. The emergent themes identified in this study are described below.

## Barriers to Help-Seeking and Prioritizing Getting Through the Assessment Period over Mental Health

1. Failure to recognize a problem existed at the time

Participants were unable to recognize symptoms they experienced as suggestive of a deterioration in mental well-being or the onset of a mental illness at the time of high-stake assessments.

life started slowly turning grey, before it started turning to black in a year’s time, but I didn’t know I was going downhill. And I just pushed myself…I was so depressed I couldn’t get out of bed. But I didn’t know much about depression then…it’s something you study but once you go through it, it’s very different. And I had severe depression. At times, I thought about ending my life. I was angry at God, at religion, at medicine as well. At everything.

Even for those with a formal medical diagnosis, participants remained surprised they had actually suffered from a mental health disorder.

I never really came across it [anxiety] before, I mean even the idea of mental health was still slightly new to me…I’d learned academically about anxiety but never realized that experience is totally different from reading about it and actually having it.

2. Denial that a problem exists

Participants reported instances where they were aware of a problem existing but they dismissed or denied it in order to continue with their studies.

I was aware. I knew what I was feeling, still I chose to ignore it and I let it get that bad before I think I even acknowledged that there was a problem.

Furthermore, participants believed they had no other choice but to ignore any problems in order to get through their studies.

I was feeling low in mood and I was mentally exhausted…I just carried on…I ain’t got another option, have I…I didn’t tell the medical school nor did I tell anyone, I didn’t tell my parents.

3. Normalization of symptoms or situation

Participants attempted to normalize the way they felt as a normal reaction to the stress of assessments, even though they were likely to be consistent with psychological distress.

I think I felt, yeah it was normal to be nervous, normal to have chest pain…it’s normal to have abdominal discomfort because it’s just how we feel when you’re nervous, isn’t it?

Participants also believed peers experienced the same or worse as them in the process of normalizing their experiences.

It was just like, loads of people have things going on and loads of people have things to deal with.

Participants believed there was no opportunity to seek help for their mental health problems since the workload to prepare for assessments took up all of their available time.

Then I was scared…I was just crying. I couldn’t sleep, I lost so much weight…I was planning to see pastoral…I just didn’t have the time, I just didn’t know when to fit that in with all of this stuff.

That [exams] is what I focused on, yes. The actual mood I didn’t appreciate was that bad, well I knew it was bad but my focus was on the exams so much, I knew it was bad but I left it…it wasn’t the priority.

Participants also identified geography as a barrier to seeking help since some individuals had limited opportunities to access support at convenient times when on placement.

I did try to contact pastoral services, but because I was in XXXXX I asked for a Friday appointment because obviously I’d be back [at medical school]… that [Friday] was the only day they weren’t open…

4. Perceptions about self, others and medical school

Participants believed they were individuals who could cope with their problems.

I feel like I knew that I wasn’t enjoying myself and perhaps I was depressed and stuff. But I didn’t do anything about it. And that’s sort of a regret, it’s something I wish I’d sorted out…I’ve always felt that I was quite, I handle pressure quite well…

Participants reported feeling that other students’ problems were more important than their own, and that others would be unsympathetic to their issues or fail to understand them.

They [family] were busy sorting things at home, really…and to be honest, I didn’t really ask for it [support]. Because, obviously, like there were other more important things than me going on.

my clinical partner was just like, you’re lazy, you’re unmotivated…Consultants…were like...you’re being lazy and unmotivated! Sick to death of hearing it by the end of it, but I thought maybe I was being lazy and unmotivated, because, if everyone says it, it must be true, right?!

Negative perceptions about the medical school were commonly reported. Students held negative perceptions, for example, about the school’s response to psychological distress or mental health problems and suggested the medical school offered little or no support for meeting their needs.

I didn’t think there was anyone that I could obviously go to, to say look “I don’t think I’m coping.” Because I wasn’t aware of anyone that was available.

I do put my walls up very quickly and if there is an ounce of insincerity or like they are just doing it [giving support] to be tokenistic, it pushes you further away.

5. Symptoms of mental distress or illness

Given participants were unable to recognize symptoms of psychological distress or a mental health disorder, symptoms such as loss of motivation, low mood and suicidal thoughts were clearly identifiable barriers to seeking help.

I think the most…the biggest factor was that I stopped enjoying medicine and I just…just lost the motivation. Yeah. It seemed like a drag going in every day, then coming home and studying and I just…I did feel quite low a lot of the time and I think that was the biggest thing.

I explained to him [personal tutor], I think I have depression. This has really affected my work. I haven’t been able to get in touch with you because I think; when you’re depressed, it’s just…you don’t really want any contact with anyone. The best thing that I wanted to do was be in my room and sleep.

6. Fear of stigmatization

A fear of being stigmatized by others as well as the medical school prevented participants from disclosing any psychological distress around the time of assessment.

I was just scared of the stigma attached to it [depression]…I thought people would look at me differently

well people said it to me mostly, “you’re not allowed to be a doctor if you suffer from depression”

The threat of reprisal from medical school among participants prevented individuals from any disclosure due a fear that faculty may use their problems as evidence against them after any potential failure at assessment.

I think a lot of people have this fear about saying something because it might be used against them if they go to an appeal...

If the med school offered any support, I don’t think I listened obviously. Because I just thought it was another way of putting me back on their radar, if you like…And I just wanted to stay away from, like, the faculty as much as possible and have the barest minimum contact with them.

Any future repercussions from a label of psychological distress or mental health disorder also prevented participants from disclosing any difficulty around the time of assessment.

and he [person tutor] goes, “well, why haven’t you told us about this [depression] sooner?”…I was like, “well I don’t want to give you a reason to, you know, put me in front of the Academic Progress Committee and say well, psychologically, you’re unsuitable to be a doctor.”

## Drivers for Help-Seeking and Prioritizing Well-being over Getting Through the Assessment

Participants’ help-seeking behavior was often slow and recognition for the need to seek help came as a last resort but there were specific drivers for accessing support.

1. Recognition that mental health is important

Participants who gave some priority to their own mental health and retained awareness about the impact of their “problem” on their academic performance or their quality of life were able to access help.

I think I have depression. This is really affecting my work…I got in touch with my GP as well. He arranged for counselling…I took time from university…I came back the following year and did a couple of months. I was feeling worse. So I got in contact with the university, they arranged for me to see Occupational Health…that’s where my medication started…

Participants who recognized appropriate help-seeking behavior as a personal as well as a professional duty were also able to seek help.

I had almost taken on this idea of being easy on myself…being a lot more compassionate to myself, as well as the sort of knowledge that this was something bigger and if I had just left this stress to go on and pass my exams, I could end up in suicide...

Participants with significant others around them to recognize the impact of psychological distress or a mental health disorder were also able to access help appropriately.

I didn’t really know I was going through such a hard time, but my boyfriend did, and my friend…she knew that I was really anxious…it started getting out of control.

2. Student feels that there is someone in whom they can trust to give them support: trust

A good relationship with a healthcare professional or member of staff at the medical school such as personal tutor, lecturer, or administrative staff empowered participants to seek help when necessary.

I went to see a very, very, very good GP…I was so sensitive about it I was, like, I need to find someone who knows mental health at least a little bit and then I found her…and just having her listen in itself was very healing…and she referred me to a counsellor…

If you failed the qualifiers you had to go see pastoral services. So I went to see Dr XXXX and she was really, really nice, so I told her everything that had happened, I started crying to her as well…she was more reassuring to me

3. Symptoms of psychological distress or a mental health disorder

Overt symptoms of psychological distress or a mental health disorder were a driver for participants seeking help.

Then I started getting really worried because I started becoming not myself at all and started becoming abnormal and having really weird thoughts. So I went to the GP and thought “I need help”… I couldn’t study, I started hearing weird things, and feeling like I wasn’t normal.

Ah, God, I almost wanted to kill myself… …when someone gets into a point of wanting to kill him or herself, it’s actually quite bad because it shows that…like, “I don’t want to live anymore. I’m totally down, I don’t like myself anymore.” That is scary.

On occasion the teaching staff at medical school identified participants’ need to access help.

I didn’t want to go in. And then the consultant got me into trouble with the medical school…and then I went to see Dr XXXX and I told her…then bells started ringing that this is really affecting me, and she told me why don’t you seek some help, go to the GP? So went to the GP then I found out about counselling.

## Discussion

There are a number of established factors that influence students’ help-seeking behavior at medical school such as a fear of stigmatization [1, 2, 13, 21–23], normalization of symptoms and situation [1, 2, 12, 24, 25] and various held perceptions by individuals of themselves, others and the medical school [1, 13, 23, 26]. The findings from this study confirm these factors as influencers on the decision-making process of students’ help-seeking behavior, with the denial of a mental health disorder [23, 24] being a key determinant on whether individuals actually seek help when in distress. In particular, this study suggests that students’ lack of awareness to their own mental health, as well as a lack of response to any awareness among those who retain it, is an important barrier to seeking help despite the support system in place to prevent or detect mental health problems among students at medical school.

Despite the plethora of interventions described, designed, developed, and delivered in medical schools for raising awareness about barriers for students to demonstrate appropriate help-seeking behaviors [23], this study suggests many students are still suffering in silence with psychological distress and mental health disorders, especially at time of assessment.

One of the interesting findings in terms of the drivers identified in this study was the fact that students who had established a trusting relationship with someone at the medical school better identified the need to seek help and access support. Previous studies have reported the importance of continuity of care from the medical school in providing a supportive and stable environment for students [27]. Given medical school curricula are split between early years and later years and medical students are dispersed at various sites along their training, establishing continuity of care remains challenging. Nevertheless, in the context of remediation, a dedicated team who support students who have failed at assessment across the whole course may be in a position to establish such a trusting and stable relationship. Clearly this would be aligned with a wall-gardened approach for remediation as called for by many in the assessment community [28].

There are a number of well-described models of behavior prediction [29, 30] with evidence of predictive value for intention and behavior action [31, 32]. However, many of the models are specific to simple choices or development from contexts outside of medical education [33–35]. There remain no models of behavioral intention or action that specifically predict student decision-making in the setting of psychological distress around time of assessment. There is a large body of evidence demonstrating the influence of assessment on driving student-learning behavior [36] however this study suggests assessment also drives student help-seeking behavior as well. The findings from this study are the first to suggest assessment negatively drives student help-seeking behavior and the prospect of preparing for high-stakes examinations exerts sufficient influence on students such that they prioritize the assessment over their personal well-being.

Although the participants included in the study were students with a history of failure at assessment and may not be representative of the wider student body, their choices in terms of prioritizing mental health over preparing for re-sits, are likely to be identical to those for individuals at other institutions who find themselves in similar circumstances. Furthermore, this study confirms the complex decision-making challenges for students when deliberating over whether to seek help in the event of a deterioration in mental health and the further damaging effects of prioritizing getting through assessment above all else. This is one of the few studies that explored the barriers students face when considering seeking help for psychological distress or a mental health disorder using a qualitative methodology and one-to-one personal interview.

That said there were a number of limitations to this study. Firstly, by expecting mental ill-health to be disclosed naturally by participants, some individuals who actually experienced mental health problems but chose not to divulge in the absence of direct questioning may have been missed from the wider sample of students. Furthermore, participants in this study had failed their final professional examination and were undergoing a period of remediation; therefore, students who had sought help for mental health problems at appropriate times and avoided failure at assessment were also excluded from the study. The inclusion criteria applied to the transcripts identified students who discussed a history of psychological distress and mental health symptoms but whether these symptoms would meet operational criteria for a mental health disorder is beyond the scope of this paper. Finally, emergent themes from the interviews were not triangulated with data from medical school records or testimonies from faculty staff who supported students through times of difficulty and failure at assessment.

There is increasing awareness among medical schools about the importance of prioritizing mental well-being and preventing mental health disorders [23]. A number of medical schools are investing in novel approaches and services to help students overcome known barriers to treatment [2, 23, 26]; however, evidence for effectiveness is lacking. The findings from this study confirm an integrated and inter-professional approach targeted at students at risk of psychological distress or a mental health disorder is necessary to overcome lack of self-awareness about their own situation and symptoms students experience when suffering from such problems. Many current models of student support are designed for students who retain or demonstrate a sufficient level of understanding and insight into their problems. Nevertheless, this study confirms students may lack the capacity to recognize their ill-being or illness in the first place. Therefore an integrated approach is necessary, which faithfully helps educators to work through all the possible barriers and drivers that influence students’ help-seeking behavior when planning prevention strategies for students.

Finding new and effective ways to encourage all students to regularly monitor their own well-being and recognize the signs of deteriorating health, particularly around times of high-stakes assessment, should be a priority for medical educators. The most effective strategies are likely to be those where students take ownership and responsibility for their health and well-being, including taking appropriate help-seeking action [37]. This challenge will be particularly difficult since medical students are in theory training to recognize symptoms of mental illness in others, yet the very same individuals seem to remain oblivious to illness within themselves.
